# Jericho open resistive data logger: An open-source modular weather station and monitoring system for long-term solar photovoltaic outdoor experimentation

**DOI:** 10.1016/j.ohx.2025.e00720

**Published:** 2025-11-07

**Authors:** Koami Soulemane Hayibo, Joshua M. Pearce

**Affiliations:** a Department of Electrical & Computer Engineering, Western University, London, ON, Canada; b Ivey Business School, Western University, London, ON, Canada

**Keywords:** Resistive data logger (RDL), Environmental monitoring, PV monitoring, Field sensor validation, Raspberry Pi, Reproducibility, Open hardware, Energy monitoring

## Abstract

Environmental and energy production monitoring systems not only provide data acquisition (DAQ) but now supervisory control and data acquisition (SCADA) for both meteorological and solar photovoltaic (PV) research. DIY systems are often not robust enough for research and proprietary systems are often economically prohibitive. The Jericho Open Resistive Data Logger (RDL) platform bridges this gap between low-cost DIY devices and high-cost proprietary DAQs. It integrates a custom RDL, Arduino Nano, modular I^2^C expansion, and a Raspberry Pi for edge processing into a robust, open-source platform. Supporting multiple sensor protocols (analog, digital, resistive, I^2^C, SDI-12, and USB) and long-distance wired transmission, the system enables reproducible, research-grade data collection at less than half of the cost of proprietary stations. Statistical comparison of irradiance, relative humidity and temperature and wind speed were bench marked against a proprietary system and found to be well within acceptable differences for validation although wind speed was found to have the highest deviation. Two independent open-source units confirm excellent inter-device repeatability across all measured variables. By combining environmental and PV monitoring within a unified platform, Jericho Open RDL provides an accessible and adaptable solution for distributed renewable energy and environmental research.

## Graphical abstract

**Figure:**
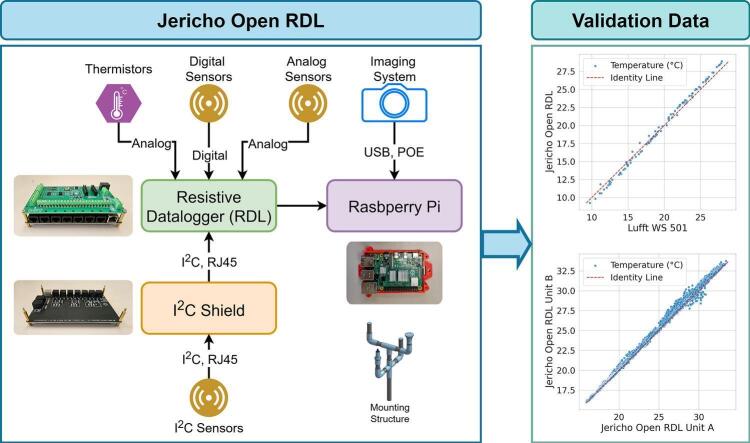

Specifications table

**Table:** 

Hardware name	Jericho Open Resistive Data Logger (JOR)
**Subject area**	• Environmental monitoring • Renewable energy systems (PV, floating PV, agrivoltaics) • Data acquisition and sensor networks
**Hardware type**	• Field measurements and sensors • Data acquisition (DAQ) system • Environmental and energy monitoring platform
**Closest commercial analog**	Onset HOBO RX3004-SYS-KIT-813 (CAD$4010.42) – No Imaging Systems
**Open source license**	Hardware: MIT Software: GNU GPL 3.0 Documentation: CC-BY-NC-SA
**Cost of hardware**	Total: ∼CAD$ 2,821.14 Without Imaging System: ∼CAD$ 1,623.14
**Source file repository**	*https://doi.org/10.17605/OSF.IO/G7K6P*

## 1 Hardware in context

Environmental monitoring systems have evolved from simple standalone weather stations to advanced, research-grade data acquisition (DAQ) and supervisory control and data acquisition (SCADA) platforms that form the backbone of meteorological and solar photovoltaic (PV) research. Commercial systems such as the Campbell Scientific CR series and GRANITE platforms [1,2], Yokogawa GM10 [3], and Delphin Expert Logger [4], are well established for their precision, robustness, and ability to integrate diverse meteorological and energy sensors. These systems are widely deployed in utility-scale PV performance monitoring field trials, where long-term reliability and compliance with standardized sensor protocols are critical [5,6]. Despite their proven accuracy, their proprietary architecture, closed data ecosystems, high data storage expenses, high capital costs and limited flexibility can hinder adaptation to novel experimental designs or integration with unconventional sensor arrays, particularly in distributed and resource-constrained research [7].

Open-source hardware has emerged to address these barriers, offering modular and lower-cost alternatives [[8], [9], [10], [11], [12]]. Arduino-based weather stations provide basic meteorological measurements at reduced cost [[13], [14], [15]], other open-source meteorological stations have evolved in functionality and are wireless [16], while platforms such as HIGROTERM demonstrate low-cost multi-channel environmental monitoring [17]. The EnviroDIY Mayfly logger [18] and other open-source weather stations illustrate how community-driven hardware can scale toward field deployments in hydrology and agriculture [19,20]. Beyond meteorological sensing, open-source air quality monitoring systems such as the SentinAir [21,22] demonstrate modular Raspberry Pi-based architectures capable of integrating heterogeneous sensors and devices. Opengear further exemplifies open-source development for environmental data sensing [23]. These systems, however, generally lack the robustness, multiprotocol and multi-microcontroller support, and sensor diversity required for long-term, research-grade applications, particularly in PV and agrivoltaic domains [19,24].

Within renewable energy research, low-cost PV monitoring solutions have been developed using microcontrollers to measure module voltage, current, and temperature [[25], [26], [27]]. While valuable as proof-of-concept tools, these lack the environmental hardening and integration of meteorological and PV measurements required for extended operation. Specialized floating PV monitoring systems have also been reported, but these are often proprietary and narrowly tailored, limiting adaptability and reproducibility [28,29]. Similarly, agrivoltaic research has highlighted the need for synchronized monitoring of crops [[30], [31], [32]], microclimate, albedo [33] and PV performance data [5], but existing solutions typically rely on separate systems for agricultural and energy measurements, complicating deployment and analysis.

The Jericho Open RDL platform is designed to bridge this gap between low-cost DIY devices and high-cost proprietary DAQs. It integrates a custom Resistive Data Logger (RDL), Arduino Nano, modular I^2^C expansion, and a Raspberry Pi for edge processing into a robust, open-source platform. Supporting multiple sensor protocols (analog, digital, resistive, I^2^C, SDI-12, and USB) and long-distance wired transmission, the system enables reproducible, research-grade data collection at a fraction of the cost of commercial stations. By combining environmental and PV monitoring within a unified platform, Jericho Open RDL provides an accessible and adaptable solution for distributed renewable energy and environmental research.

## 2 Hardware description

The Jericho Open RDL (JOR) data acquisition system is composed of three principal subsystems: (i) the data acquisition and processing hardware, (ii) the sensor array for experimental measurement, and (iii) the embedded software framework responsible for system operation, sensor‐to‐DAQ communication, and management of local data storage.

### 2.1 Data acquisition and processing platform

The data acquisition and processing platform consists of an RDL paired with an I2C extension shield, an Arduino Nano microcontroller, a Raspberry Pi 4 single-board computer, and the necessary structural and electrical fixtures that support the central hub’s operation.

#### 2.1.1 The resistive datalogger board

**Fig. 1:**
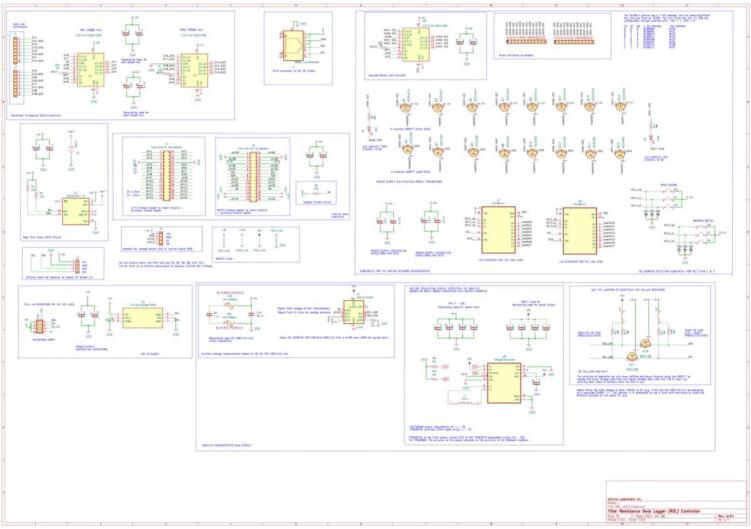
Electrical schematic of the RDL.

The RDL is a modular, open-source platform engineered for acquiring data from resistive, analog, digital, and I^2^C sensors. The hardware integrates sequential sensor communication, multiplexing, and precision analog-to-digital conversion into a scalable architecture optimized for distributed monitoring in both environmental and laboratory settings. The RDL facilitates long-distance wired communication between the sensors and the Arduino, enabling deployment in field applications where sensors must be positioned remotely from the central data hub. The electrical schematic for the RDL is presented in Fig. 1.

##### 2.1.1.1 RDL core controller

The RDL's central controller, an Arduino Nano, controls sensor energization, channel selection, data acquisition, and communication with peripheral modules. PWM digital outputs drive the multiplexer banks. Pins D10 and D11 govern the analog-sensor and thermistor multiplexer enable lines, while D3 to D5 specify active channels. Multiplexer outputs route to analog pins A0 and A1 for digitization via the Nano’s internal 10-bit ADC or the external 16-bit ADS1115. A DS3231 RTC on the I2C bus delivers precise timestamps, backed by a button-cell battery. I2C frequency is reduced from 100 kHz to 490 Hz to avoid signal degradation due to high bus capacitance and minimize EM interference over long cabling.

##### 2.1.1.2 Temperature sensor acquisition and multiplexing

Temperature acquisition and multiplexing leverage two CD74HC4051 analog multiplexers, enabling up to eight thermistors. Both high and low sides of each thermistor are switched in series to reduce interference and leakage over cable runs up to 30 m. Outputs route to pin A0 for digitization, and firmware compensates for the 140 Ω total multiplexer on-resistance. Each voltage divider uses a precision SMD reference resistor for stable measurements.

##### 2.1.1.3 Analog sensors acquisition and multiplexing

Eight 0–5 V analog channels are switched by CD74HC4051 multiplexers, while power switching uses P-channel AO3401A and N-channel AO3406 MOSFET pairs controlled via PCF8574 GPIO expanders over I^2^C, synchronized with multiplexer addressing. Outputs feed analog pin A1, and GPIO expanders allow temporary software mapping of analog lines to digital functions for enhanced flexibility.

##### 2.1.1.4 I^2^C shield

**Fig. 2:**
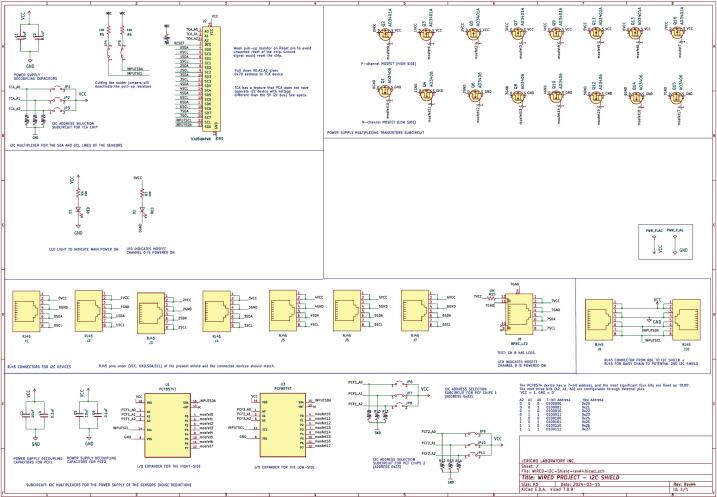
Electrical Schematic of the I^2^C shield board.

An external I^2^C shield extends digital acquisition capacity and selective channel power. Sensors connect via RJ45 over twisted-pair CAT5–CAT7 to mitigate noise. An LTC4311 I^2^C extender on the main board strengthens the bus for daisy-chaining up to three shields. Each shield uses a TCA9548 multiplexer for address isolation and PCF8574-MOSFET pairs for channel power. Dual RJ45 ports support modular, scalable deployments (Fig. 2).

#### 2.1.2 Raspberry Pi single board computer (RPi-SBC)

A Raspberry Pi 4B serves as the central processing and data management hub. It receives data from the Nano via USB, appends NTP-synchronized timestamps, and organizes files through a multi-stage directory system to prevent corruption. Python-based daemons manage logging, cloud synchronization, imaging acquisition, and system status monitoring. Services automatically restart on boot or failure, enabling continuous operation with minimal user intervention. Remote access is supported via RustDesk [34], and active cooling ensures stability in hot environments.

#### 2.1.3 System enclosure and support infrastructure

All components are enclosed in a waterproof ABS IP65 junction box rated for –40 °C to +40 °C (internally up to 100 °C). A downward-facing rubber gasket and cable glands prevent water ingress. An internal ABS mounting plate secures the RDL, I^2^C shield, and Pi, while a power distribution bar on the door supplies six surge-protected outlets (1000 J). Internal cabling is managed with adhesive fixtures and tie wraps, silica gel packs absorb moisture, and M25 glands with foam inserts seal unused ports.

### 2.2 Sensor arrays and measurement capabilities

The JOR DAQ is designed as a flexible acquisition platform, capable of interfacing with analog, digital, I^2^C, and USB sensors. Sensors are connected via standardized RJ45 ports or multiplexer channels, ensuring interoperability and long-cable deployment. Below, the weather and PV monitoring sensors integrated into the system are described individually.

#### 2.2.1 Weather station

##### 2.2.1.1 Air Temperature and Humidity

**Fig. 3:**
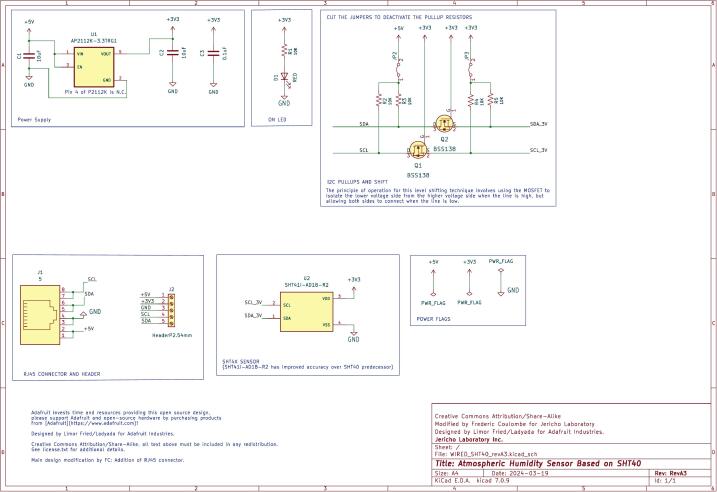
Electrical schematic of the air temperature and humidity sensor (SHT41) board.

The air temperature and humidity sensor is based on the Sensirion SHT41I-AD1B-R2 digital sensor. It offers temperature and humidity accuracies of ±0.3 °C and ±2 %, respectively, meeting World Meteorological Organization standards [35]. The board incorporates an RJ45 connector for direct integration into the RDL system, a low-dropout regulator for clean 3.3 V operation, and a status LED for deployment checks. The PCB layout includes a central cutout to improve airflow when mounted in a radiation shield [36]. (See Fig. 3)

##### 2.2.1.2 Solar irradiation

Solar irradiance is measured with the Apogee SP-421 silicon-cell pyranometer [37], which communicates via SDI-12. Its spectral response (360–1120 nm) matches crystalline silicon PV modules, making it well suited for PV yield studies [38]. The sensor includes a low-power heater for dew/frost mitigation, controllable in software, and is deployed on a CM226 stand with bubble level for horizontal alignment.

##### 2.2.1.3 Wind Speed

Wind speed is monitored using a three-cup anemometer (PN 1528–1328-ND). It provides an analog voltage output proportional to wind speed across a 0–50 m/s range. Since its supply requirement (7–24 V) exceeds the native 5 V rail, a DC–DC boost converter is used for power conditioning. Output is routed through the analog multiplexer for acquisition, supporting both 10-bit and 16-bit digitization.

#### 2.2.2 PV monitoring

##### 2.2.2.1 PV Temperature

**Fig. 4:**
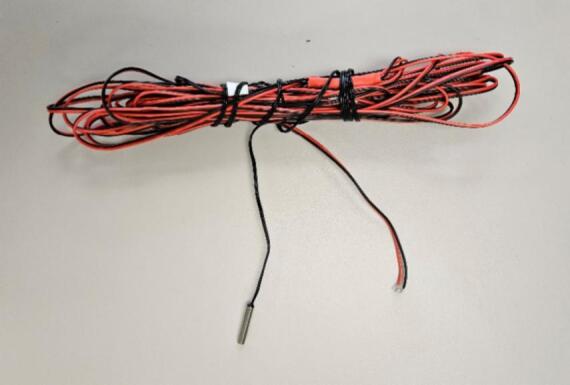
10 kΩ NTC thermistor with 30 m extension cable and waterproofed.

NTC TH-2 thermistor probes (10 kΩ at 25 °C) are used to measure PV modules temperature (Fig. 4). Probes can be extended up to 30 m without significant degradation, making them suitable for floating PV or distributed environmental monitoring. For reproducibility, probes undergo a three-point calibration (0 °C, ∼37 °C, and 100 °C) to derive Steinhart–Hart coefficients used in firmware. Only probes with identical coefficients are retained to conserve Nano memory.

##### 2.2.2.2 Visible light image

A Reolink RLC-520A IP camera provides visible-light imaging in an IP66 enclosure [39]. Though rated to −10 °C, its approximately 3 W operating consumption allows reliable use down to −40 °C in Canadian winter deployments. Low temperature operation capability was tested using dry ice. The camera is powered via Power-over-Ethernet and communicates with the Raspberry Pi through a static IP configuration. Images are captured via an HTTP polling script at user-defined intervals.

The visible light camera provides contextual imaging for PV array supervision and site surveillance. It allows visual correlation of system performance with site conditions such as snow accumulation and snow days detection [[40], [41], [42]], debris, shading, and supports remote inspection and long-term observation.

##### 2.2.2.3 Infrared image

Thermal imaging is performed using the Seek Thermal C214SPX long-wave infrared core [43], integrated into a custom ABS enclosure with a germanium window. It provides 200 × 150 resolution thermograms in CSV and colorized JPEG format, captured via USB and logged by the Raspberry Pi. USB-over-CAT adapters extend cable length, enabling flexible sensor placement. Non-uniformity correction (NUC) is handled by the onboard mechanical shutter.

In a PV experiment, the infrared camera enables non-contact thermal mapping for arrays under investigation to detect temperature-related operational issues including hotspots, shading impact, and thermal non-uniformities. The spatial thermal data complement point-based thermistor measurements, allowing a more comprehensive assessment of PV thermal behavior [44].

##### 2.2.2.4 DC Current

**Fig. 5:**
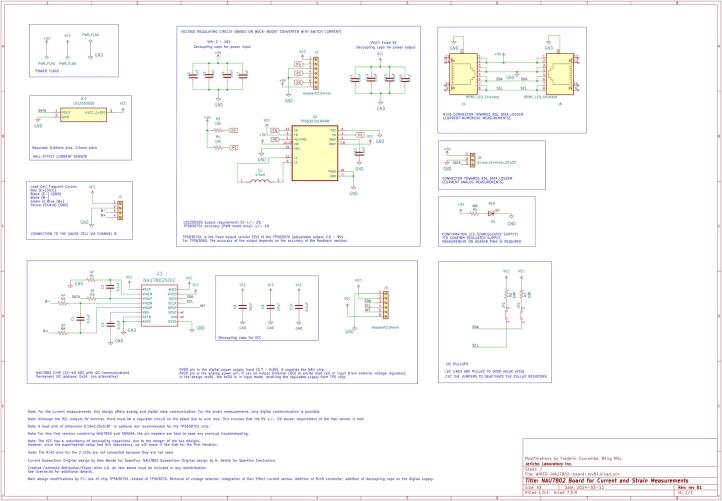
Electrical schematic of the current sensor board layout.

DC current is measured with the Tamura L01Z050S05 closed-loop Hall-effect transducer, covering ±50 A ±1 % rated accuracy. The output is digitized by a NAU7802 24-bit ADC (Fig. 5) for high-resolution measurements. A TPS630701 buck–boost regulator maintains a precise 5 V supply, and RJ45 connectors allow daisy-chaining. For stability, a nearby thermistor connected to the RDL can provide optional temperature compensation.

### 2.3 Software architecture

#### 2.3.1 Raspberry Pi software description

The Raspberry Pi runs 64-bit Pi OS and executes Python 3 scripts alongside the Arduino firmware. It handles USB serial input, image capture, system monitoring, and data organization. Incoming serial packets from the Nano are timestamped via NTP and written to an in-progress directory. Completed datasets are atomically moved to a to-sync directory to prevent access to partial files. A watchdog timer detects serial stalls: upon timeout, the logging script exits gracefully and is relaunched by *systemd* for automated recovery.

Infrared outputs are logged as CSV arrays, and false-color JPGs are generated at configurable intervals. The visible-light camera captures independent JPEG snapshots. Both pipelines adhere to the in-progress/to-sync staging hierarchy, ensuring synchronized file handling across sensors.

**Fig. 6:**
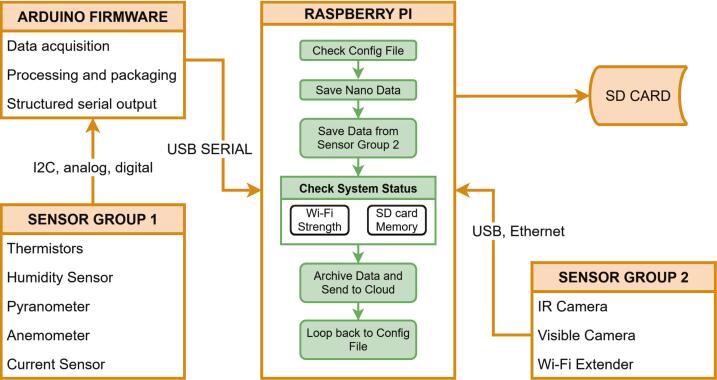
Jericho Open RDL software workflow diagram.

Real-time metrics; including Wi-Fi signal strength, SD-card capacity, CPU load, and device temperature; are tracked continuously. Exceeding predefined thresholds triggers safeguards: restarting services, suspending nonessential processes, or controlled reboot. All events are logged for traceability. The architecture, summarized in Fig. 6, provides a modular framework in which additional sensors or services can be incorporated with minimal change to existing processes.

#### 2.3.2 Arduino Nano firmware

**Fig. 7:**
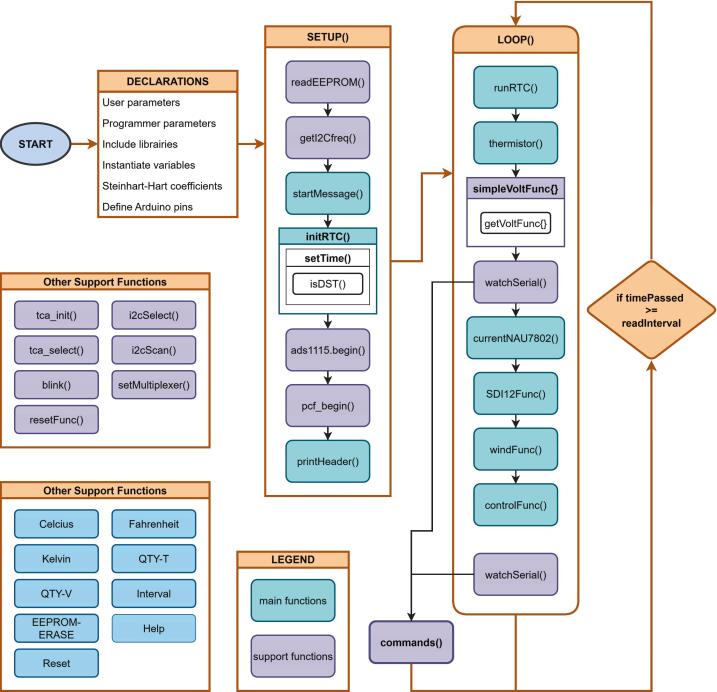
RDL Arduino Nano firmware diagram.

Firmware on the Nano comprises variable declarations, initialization, and a continuous acquisition loop. User parameters (i.e., sampling interval, Steinhart–Hart coefficients, sensor-activation flags) and programmer parameters (i.e., pin maps, bus addresses) reside in EEPROM and load at boot. Initialization synchronizes the RTC, configures ADCs and multiplexers, and generates a dynamic header documenting the active configuration. In the main loop, elapsed intervals trigger enabled routines, appending processed measurements to a structured serial record sent to the Raspberry Pi. A lightweight serial command interpreter allows on-the-fly adjustment of intervals, units, and active channels, and supports EEPROM resets, reducing the need for recompilation in the field (see Fig. 7).

### 2.4 Potential applications

• The JOR DAQ allows assessment of photovoltaic performance and validation of energy models. The ability to jointly monitor environmental and electrical variables makes the platform well suited to agrivoltaic research [20,45] (e.g., crop–microclimate interactions with panel efficiency, soil characteristics measurement) and floating PV installations [28,46,47](e.g., where water–atmosphere dynamics influence energy yield and system reliability).
• The sensor arrays can measure simultaneously multiple weather data (temperature, humidity, wind, irradiance), supporting applications from microclimate mapping to long-term ecosystem studies.
• Integrated system diagnostics, and reporting features allow long-term, unattended deployments in isolated or logistically difficult locations, reducing dependence on continuous human oversight.
•  The hardware and software architecture accommodates straightforward integration of new sensors and transducers, facilitating adaptation to novel laboratory or field applications beyond the original environmental and energy focus.

## 3 Design files summary

### 3.1 3D Parts

All 3D parts in Table 1 are parts used as support for the components in the design (cameras and sensors stands). The parts are printed on an open-source Prusa i3 MK3S 3-D printer using poly(ethylene) terephthalate glycol (PETG) filament with the print parameters found in Table 2. It should be noted that any RepRap-class open source fused filament fabrication-based desktop 3-D printer could be used [[48], [49], [50]] and for outdoor components acrylonitrile styrene acrylate (ASA) is recommended for its superior UV resistance and applications in previous meteorological systems [36]. The STL and 3MF files have been included in the repository to allow adjustments by users. The raspberry Pi top and bottom covers are existing open-source designs; therefore, copies were placed in the repository under the original open-source license (CC-BY-4.0) [51].

**Table 1:** List of design files and codes.

Design file name	File type	Open-source license	Location of the file
Angle_Connector	CAD File (STL, 3MF)	MIT	https://doi.org/10.17605/OSF.IO/G7K6P
Camera_Holder	CAD File (STL, 3MF)	MIT	https://doi.org/10.17605/OSF.IO/G7K6P
Camera_Nut	CAD File (STL, 3MF)	MIT	https://doi.org/10.17605/OSF.IO/G7K6P
Camera_Stand	CAD File (STL, 3MF)	MIT	https://doi.org/10.17605/OSF.IO/G7K6P
Camera_Support_Angle	CAD File (STL, 3MF)	MIT	https://doi.org/10.17605/OSF.IO/G7K6P
Cross_Connector	CAD File (STL, 3MF)	MIT	https://doi.org/10.17605/OSF.IO/G7K6P
Humidity_Sensor_Adapter	CAD File (STL, 3MF)	MIT	https://doi.org/10.17605/OSF.IO/G7K6P
Humidity_Sensor_Plates	CAD File (STL, 3MF)	MIT	https://doi.org/10.17605/OSF.IO/G7K6P
Humidity_Sensor_Stand	CAD File (STL, 3MF)	MIT	https://doi.org/10.17605/OSF.IO/G7K6P
Humidity_Sensor_Top_Plate	CAD File (STL, 3MF)	MIT	https://doi.org/10.17605/OSF.IO/G7K6P
IR_Camera_Support	CAD File (STL, 3MF)	MIT	https://doi.org/10.17605/OSF.IO/G7K6P
Pipe_Terminator	CAD File (STL, 3MF)	MIT	https://doi.org/10.17605/OSF.IO/G7K6P
Raspberry_Pi_Top_Cover	CAD File (STL)	CC-BY-4.0	https://doi.org/10.17605/OSF.IO/G7K6P
Raspberry_Pi_Bottom_Cover	CAD File (STL)	CC-BY-4.0	https://doi.org/10.17605/OSF.IO/G7K6P
Sensor_Support	CAD File (STL, 3MF)	MIT	https://doi.org/10.17605/OSF.IO/G7K6P
T_Connector	CAD File (STL, 3MF)	MIT	https://doi.org/10.17605/OSF.IO/G7K6P
VL_Camera_Support	CAD File (STL, 3MF)	MIT	https://doi.org/10.17605/OSF.IO/G7K6P
Wind_Speed_Sensor_Plate	CAD File (STL, 3MF)	MIT	https://doi.org/10.17605/OSF.IO/G7K6P
RDL_main.zip	Arduino Codes (*.ino)	GNU GPL 3.0	https://doi.org/10.17605/OSF.IO/G7K6P
EEPROM-initialization.zip	Arduino Codes (*.ino)	GNU GPL 3.0	https://doi.org/10.17605/OSF.IO/G7K6P
Arduino-libraries.zip	Arduino Libraries (C++)	GNU GPL 3.0	https://doi.org/10.17605/OSF.IO/G7K6P
Python-Scripts.zip	Raspberry Pi Python (*.py) and bash Codes (*.sh)	GNU GPL 3.0	https://doi.org/10.17605/OSF.IO/G7K6P
Validation_data.zip	CSV	CC-BY-NC-SA	https://doi.org/10.17605/OSF.IO/G7K6P
Schematics.pdf	Electrical Schematics	CC-BY-NC-SA	https://doi.org/10.17605/OSF.IO/G7K6P

**Table 2:** Parameters used for the 3D printed parts.

Parameter	Value
Printer	Prusa i3 MK3S
Filament	PETG
Nozzle size	0.6 mm
Layer height	0.3 mm
Print temperature	240 °C
Bed temperature	85 °C
Support	On build plate only

### 3.2 Software code

The software codes include the RDL Arduino codes, Arduino libraries, python, and bash codes for the Raspberry Pi. The Arduino codes contain all codes necessary to run the RDL and must be uploaded to an Arduino Nano. The code is adjustable for the user’s needs. The Python codes support the data collection and storage as well as the main hub’s continuous operation.

### 3.3 Validation data

The validation data contains the files recorded from the hardware and those from the commercial hardware. These data files are used to validate the performance of the proposed hardware against a commercial counterpart and analyze the repeatability of the proposed hardware’s operation.

### 3.4 Schematics

The schematics comprises the electrical layout of the different boards used in the hardware design, including the RDL, I^2^C shield, current sensor, and humidity sensor.

## 4 Bill of materials summary

Table 3 contains the bill of materials summary.

**Table 3:** Cost summary of the materials used to assemble the hardware.

Designators	Component	Number	Cost per unit – CAD$	Total cost – CAD$	Source of materials	Material type
MH7; MH8; MH15	RDL	1	119.00	119.00	https://jericholab.com/products/	PCB
MH13	I2C Shield	1	79.00	79.00	https://jericholab.com/products/	PCB
MH1; MH2;	Main enclosure	1	99.00	99.00	https://www.amazon.ca/LMioEtool-Waterproof-Dustproof-Enclosure-Dimension/dp/B0CC8LW3GM	ABS
MH14	RJ45 Connector	1	6.80	6.80	https://www.amazon.ca/StarTech-com-Patch-Cable-Ethernet-Snagless/dp/B06Y651SKV/	Electronics
MH16	Raspberry Pi power supply	1	10.95	10.95	https://www.pishop.ca/product/raspberry-pi-15w-power-supply-us-black/	Electronics
MH5	Raspberry Pi	1	76.95	76.95	https://www.pishop.ca/product/raspberry-pi-4-model-b-4gb/	Electronics
MH12	Raspberry Pi fan	1	11.98	11.98	https://www.amazon.ca/GeeekPi-Raspberry-Radiator-Aluminum-Heatsinks/dp/B07C9C99RM/	Electronics
MH6	SD Card	1	13.95	13.95	https://www.pishop.ca/product/raspberry-pi-sd-card-32gb/	Electronics
MH10	USB Hub	1	21.99	21.99	https://www.amazon.ca/atolla-Aluminum-Splitter-Ports-Windows/dp/B09NHQXCSP	Electronics
MH9	Wi-Fi extender	1	9.00	9.00	https://www.amazon.ca/SmartQ-Expander-Transfer-Splitter-Compatible/dp/B09PTVSMLB/?th = 1	Electronics
MH19, MH21	Cable glands pack	5	0.37	1.83	https://www.amazon.ca/dp/B09V7HZPQ6/	Nylon
MH22	Power bar	1	29.59	29.59	https://www.amazon.ca/Waterproof-Weatherproof-Protector-Protection-Shockproof/dp/B0BL79QGZZ/	Electrical
MH20	Power bar plug	1	6.79	6.79	https://www.rona.ca/en/product/eaton-15-amp-125-volt-3-wire-yellow-grounding-plug-4867-sp-l2-04805786	Electrical
MH17	Power bar plug enclosure	1	6.60	6.60	https://www.amazon.ca/Waterproof-Restmo-Weatherproof-Electrical-Connections/dp/B08CMSXWGF/	Rubber
−	Silica gel pack	2	0.10	0.20	https://www.amazon.ca/Sukh-130PCS-2Gram-Silica-Packets/dp/B0CGHY9VWC/	Silica
CS1 − CS6	Current sensor with ABS enclosure	1	109.00	109.00	https://jericholab.com/products/	PCB
RH1; RH5	Humidity sensor	1	29.00	29.00	https://jericholab.com/products/	PCB
−	Thermistors	8	13.80	110.40	https://jericholab.com/products/	Electronics
RH2	M3 standoff pack plastic	100	0.02	2.34	https://www.amazon.ca/Standoff-Motherboard-Assortment-M3-JUNCHEN/dp/B0BX9G29PD/	Nylon
MH18	M3 standoff pack metal	30	0.14	4.10	https://www.amazon.ca/MECCANIXITY-Male-Female-Stainless-Assortment-Raspberry/dp/B0D6WRMLCC/	Brass
MH18; RH2	M3 screws	30	0.04	1.33	https://www.amazon.ca/440pcs-Stainless-Socket-Screws-Assortment/dp/B01N3YT1OZ/	Stainless Steel
−	M6 screws	20	0.57	11.43	https://www.amazon.ca/Button-Socket-Screws-Finish-Threaded/dp/B0C463C778/?th = 1	Alloy Steel
−	M6 nuts	36	0.20	7.20	https://www.amazon.ca/uxcell-100Pcs-Stainless-M6x12x1-2 mm-Washers/dp/B0DZWVSZYR/	Stainless Steel
−	M6 wingnuts	2	0.54	1.09	https://www.amazon.ca/uxcell-Carbon-Plated-Tighten-Butterfly/dp/B07QC5CZ8C	Carbon Steel Zinc
PY1; PY2	Silicon cell pyranometer (SP-421)	1	582.62	582.62	https://www.apogeeinstruments.com/sp-421-ss-sdi-12-digital-output-silicon-cell-pyranometer/	Electronics
PY3	Mounting stand	1	105.01	105.01	https://www.scaledinstruments.com/shop/campbell-scientific/accessories-campbell-scientific/campbell-scientific-cm226-apogee-solar-sensor-mounting-stand-w-level-base/	Stainless Steel
WS1; WS2	Cup anemometer	1	69.81	69.81	https://www.digikey.ca/en/products/detail/adafruit-industries-llc/1733/5356813	Electronics
WS3	DC-DC boost converter	1	2.92	2.92	https://www.amazon.ca/JZK-Converter-Voltage-Adjustable-Transformer/dp/B094D6JPXP/	Electronics
−	IPEX Xirtex 1 in. x 10ft	1	30.49	30.49	https://www.rona.ca/en/product/xirtec-plain-end-pipe-in-white-pvc-1-in-x-10-ft-for-potable-water-022750–0068582	PVC
−	Angle bar	1	23.49	23.49	https://www.rona.ca/en/product/precision-angle-bar-galvanized-steel-perforated-slotted-48-in-l-x-1–1-2-in-w-x-5–64-in-t-142–202-63865475	Galvanized Steel
−	PETG Filament	2	22.94	45.88	https://www.amazon.ca/OVERTURE-Filament-Consumables-Dimensional-Accuracy/dp/B08BBQ2ZCJ/	PETG
IR1 − IR10	Reolink camera with ABS enclosure	1	199.00	199.00	https://jericholab.com/products/	Electronics
VL1 − VL6	Thermal camera with ABS enclosure	1	999.00	999.00	https://jericholab.com/products/	Electronics
Total				2,827.74		

The RDL, I^2^C shield, current sensor, IR camera, and visible light camera were obtained from Jericho Lab [52]. It should be noted that all the components necessary for assembling each of these sub-systems are included in the purchase from Jericho Lab.

## 5 Build instructions

The complete assembly of the JOR DAQ weather station and monitoring system is accomplished in multiple steps. Each sub-system; including the main hub, the weather station, and the PV monitoring; is assembled separately, then all subsystems are connected to the main hub before operating the DAQ. Required assembly tools:

• Screwdrivers matching the screw-head types used in MH18 and MH20.
• Drill with a 2.5 mm bit for M3 mounting holes.
• Optional 2.5 cm hole‐saw bit for cutting cable‐gland openings in MH1, if not pre-drilled.

For all outdoor-deployed enclosures, verify that:

• The housing is fully watertight with no visible leaks.
• The rubber gasket is correctly seated and free of damage.
• Cable glands are securely tightened; any unused openings are sealed with foam inserts.
• Sufficient silica gel is placed inside to absorb moisture and inhibit humidity ingress.

### 5.1 Main hub assembly

**Fig. 8:**
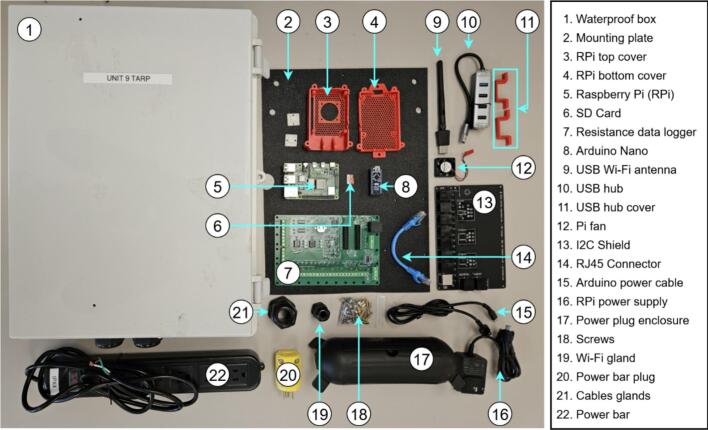
Jericho Open RDL data acquisition and processing hub.

All the components used in the main hub assembly are illustrated in Fig. 8. Components are labeled MHX, where X corresponds to the item number in Fig. 8, and the hub itself is referred to as MH.

**Fig. 9:**
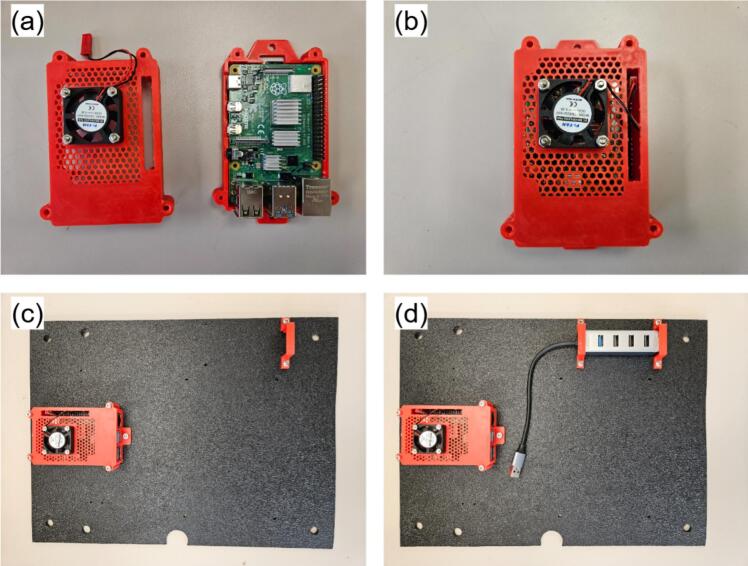
Raspberry Pi (R-Pi) and USB hub assembly: (a) R-Pi and Pi fan installed in 3D printed case, (b) Enclosed R-Pi, (c) R-Pi installation on ABS slab, and (d) USB hub installation on slab.
• Position MH5 within MH4, close with MH3 (Fig. 9a-b), and connect MH12 to MH5.
• Install the assembled Pi on MH2 using M3 screws and nuts. Mount the Pi assembly on MH2 with M3 screws and nuts, pre-drilling 2.5 mm holes at approximate locations (Fig. 9c).
• Pre-drill and install MH1 on the enclosure using M3 screws (Fig. 9c).
• Affix MH10 beneath MH11, apply a small silicone pad beneath MH11 to prevent stress fractures, then secure the second MH11 unit with screws (Fig. 9d).
b. RDL (MH7) and I2C shield (MH13)

The details of the MH assembly instructions are as follows:

a. Raspberry Pi (MH5) and USB hub (MH10)
• Insert MH8 into the SD-card slot of MH5 after loading the operating system (see Section 6).
• Secure MH12 to the exterior of MH3 using M2.5 screws and nuts (Fig. 9a).

**Fig. 10:**
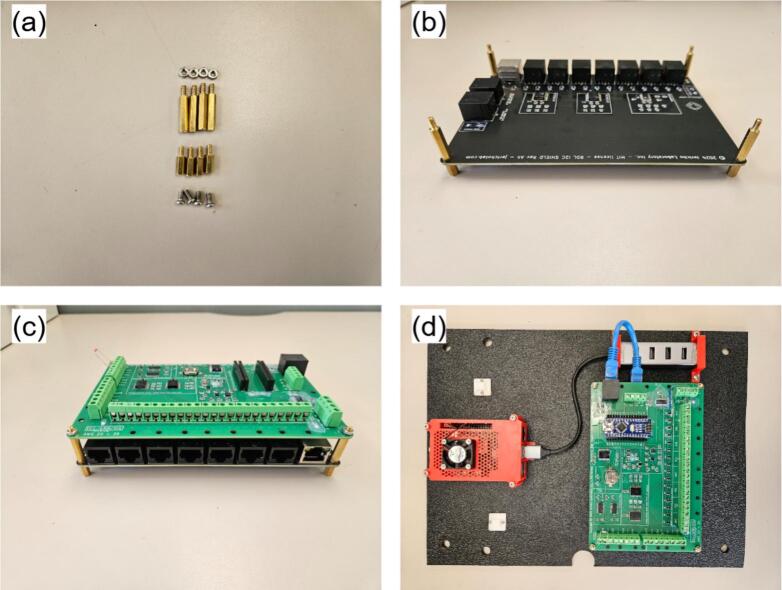
RDL assembly steps: (a) M3 screws and PCB standoffs, (b) I2C shield with M3 PCB standoffs installed, (c) RDL installed on top of shield, and (d) Assembled RDL installed on mounting plate.
• Mount MH7 atop MH13 with M3 nuts, aligning analog headers and I^2^C/RJ45 ports on the same side (Fig. 10c).
• Plug MH8 into MH7′s pin header (USB port oriented toward the board edge), connect the USB hub MH10 to MH5, and link MH7 to MH13 using cable MH14 (Fig. 10d).
c. Main enclosure (MH1) and power bar (MH22)

Assuming fully assembled boards from Jericho Lab [52] (omit component soldering):

• Position MH13 on MH2 and pre-drill four M3 mounting holes using MH13′s corner apertures as a template.
• Screw a pair of PCB standoffs at each corner of the MH13. Choose the PCB standoffs size to have enough clearance between MH13 and NH7; as well as MH13 and MH2. Install PCB standoffs at each MH13 corner (Fig. 10b).

If the power bar lacks a removable plug, procure a separate removable plug, cut off the original, and strip the conductors. Omit the first three steps if MH1 arrives with cable glands.

**Fig. 11:**
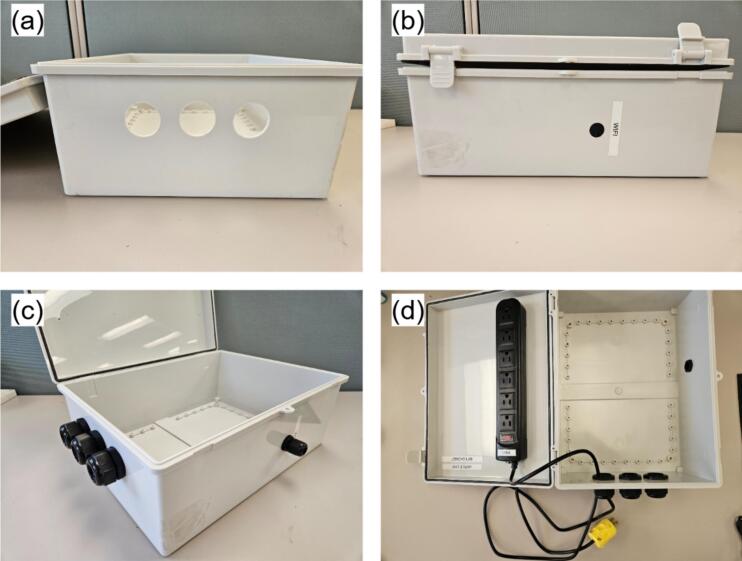
Main enclosure and power bar assembly: (a) cable glands hole, (b) Wi-Fi extender hole, (c) enclosure with mounted glands, and (d) enclosure with mounted power bar.
• Optionally, bore a side port sized for the Wi-Fi extender (MH9) if external wireless access is needed (Fig. 11b).
• Mount the MH21 to the bottom holes and MH19 to the side one (Fig. 11c).
• Pre-drill two holes (for M3 screws) in the enclosure door using the mounting holes of MH22 as guides. Secure MH22 to the door using M3 screws and nuts. Route MH22 power cord through one the holes at the bottom of MH1, then re-assemble MH20 with MH22 (Fig. 11d).
d. Main hub final assembly
• Secure MH2 within MH1 using M4 screws at the four corner mounting points (Fig. 12a). If pre-drilled holes are absent, bore them during assembly and apply silicone sealant around the exterior screws to maintain enclosure waterproofing.

• Use the 2.5 cm hole-saw to cut bottom openings in MH1 for sensor cabling, matching the required cable count (Fig. 11a).

**Fig. 12:**
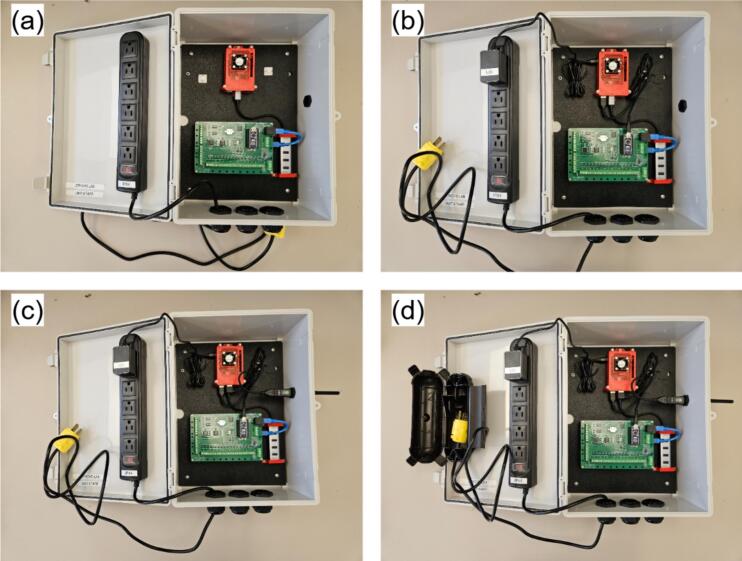
Main hub final assembly: (a) ABS mounting plate insertion, (b) Power lines connection, (c) Wi-Fi extender connection, and (d) Power plug enclosure.
• Connect MH16 to MH22 and plug its USB end into MH5. Link MH8 to MH5 using cable MH15 (Fig. 12b)
• Mount MH9 in the side gland and connect it to MH5 via USB-A cable (Fig. 12c)
• The main hub is deployment-ready pending sensor installation. Use MH17 to weatherproof the power connection (MH20) when extension cords are required (Fig. 12). Integrated mounting holes enable installation on any flat vertical surface.

### 5.2 Weather station

Weather-station sensors are assembled individually before mounting on a common stand. Following the main-hub notation, the humidity sensor, pyranometer, and wind-speed sensor are designated RH, PY, and WS, respectively.

a. Air temperature and humidity sensor

**Fig. 13:**
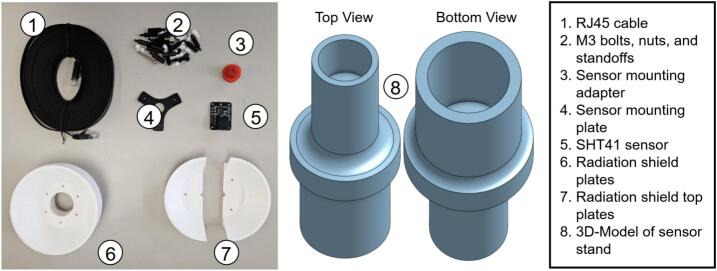
Air temperature and humidity sensor design components.

All components for the humidity sensor are illustrated in Fig. 13. RH2 must include M3 bolts (45 mm, 20 mm, and 10 mm lengths) and 2 cm M3 standoffs with nuts.

**Fig. 14:**
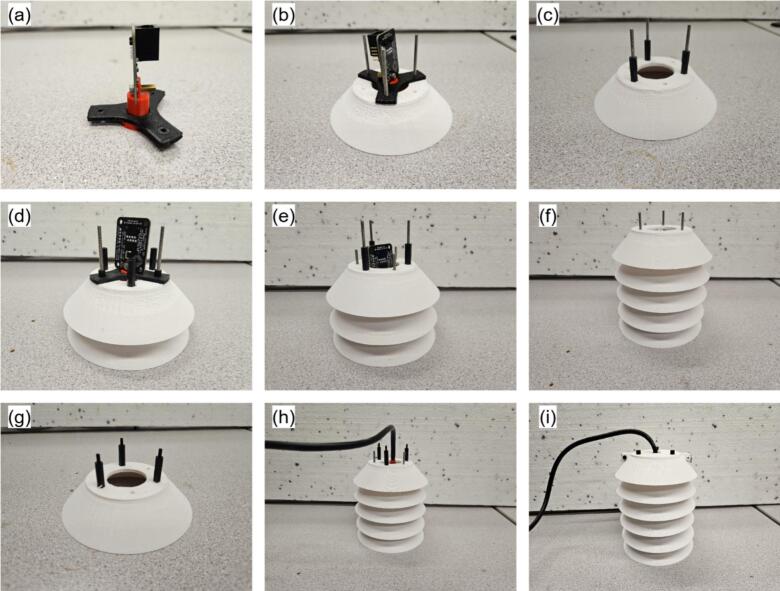
Assembly procedure of the humidity sensor.
• Align RH4 holes with one RH6 unit, insert 45 mm bolts from beneath, and fasten with nuts to mount the sensor assembly atop RH6 (Fig. 14b).
• In a second RH6 unit, insert three 45 mm bolts with one empty hole between each, securing with standoffs (Fig. 14c).
• Mount this RH6 beneath the sensor-holding unit using the vacant holes (Fig. 14d).
• Repeat the bolt-and-standoff assembly for two additional RH6 units (Fig. 14c) stacking one atop the RH6 holding the sensor, and the second on top of the first (Fig. 14e-f).
• Assemble a final RH6 with 20 mm bolts and threaded standoffs, add to the stack, and plug one end of RH1 into the RJ45 connector on RH5 (Fig. 14g-h).
• Cap the assembly with RH7, securing with nuts and 10 mm bolts at the edges. RH7 is designed for RH1 connection through its center (Fig. 14i). Apply silicone or waterproof tape to prevent water ingress at the top.
• Insert RH3 into RH8 to complete installation. The components fit tightly; additional sealant may be applied for security
b. Pyranometer
• Remove the mounting bolt from PY1 (Fig. 15a).

• Slide RH4 onto RH3 and secure RH5 with a 10 mm bolt (Fig. 14a).

**Fig. 15:**
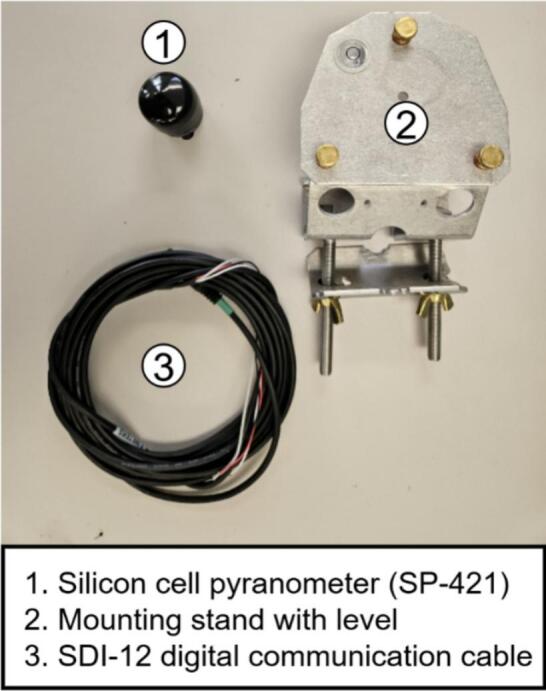
Silicon cell pyranometer (SP-421) with mounting stand and communication cable.
• Position PY1 atop PY2, aligning with the two pre-drilled holes, and secure with screws (Fig. 15b).
• Connect cable PY3 to PY1 (Fig. 15c).
c. Wind speed sensor
• Mount WS1 atop WS4 using M3 bolts and nuts.
• Connect cable WS2 to WS1.
• Solder the positive and negative leads of WS2 to WS3′s input, verifying polarity from the anemometer datasheet.
• Attach two additional output wires to WS3. (See Fig. 16.)

**Fig. 16:**
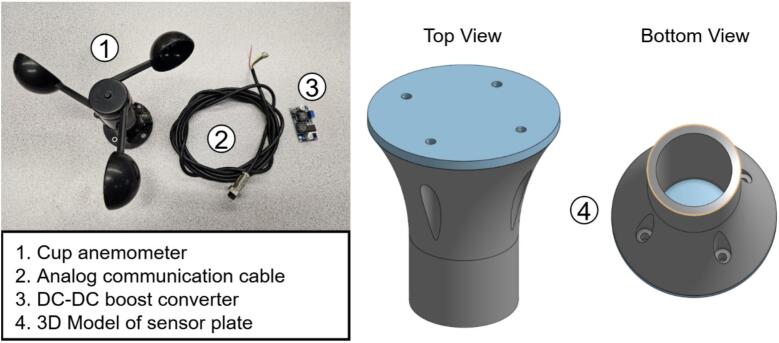
Anemometer design components.

### 5.3 PV monitoring

**Fig. 17:**
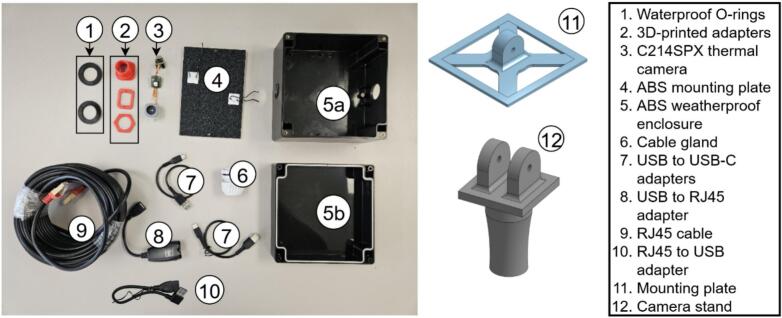
Seek Thermal C214SPX infrared thermal camera with 3D-printed parts and enclosure.

**Fig. 18:**
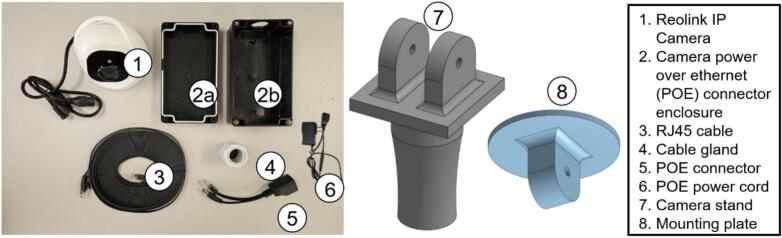
Reolink IP visible light camera with POE connector and 3D-printed parts.

**Fig. 19:**
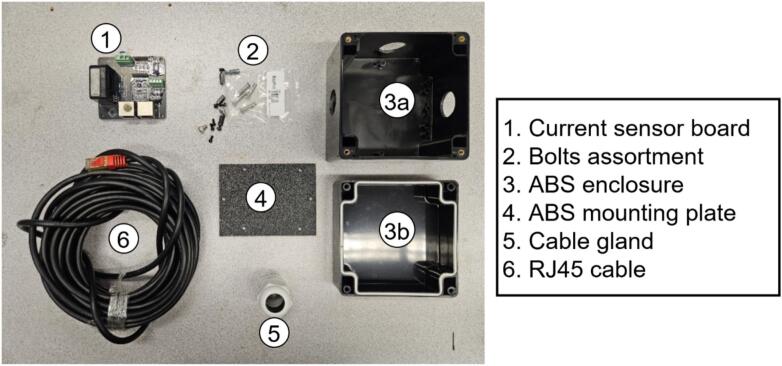
Current sensor design components.

The same naming convention as the main hub is used here. The PV monitoring sensors comprises the infrared camera (IR; Fig. 17), the visible light camera (VL; Fig. 18), and the current sensor (CS; Fig. 19). The IR and VL modules are assembled separately before co-mounting on a common support, while the CS module is typically installed in situ at the point of measurement.

a. IR Camera

**Fig. 20:**
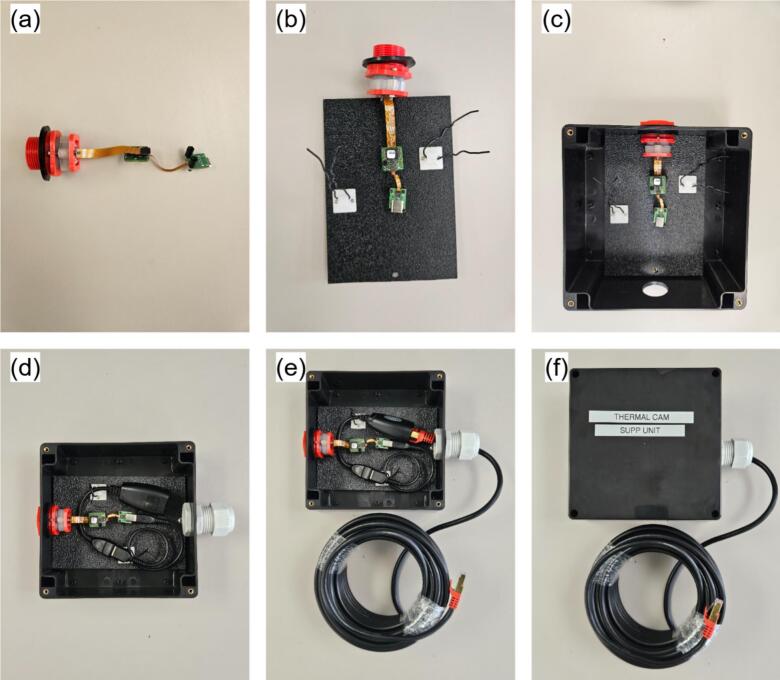
Assembly procedure of the infrared thermal camera.

The assembly of the IR Camera is displayed in Fig. 20.

• Insert the camera end of IR3 into the 3D-printed lens mount piece in IR2, slide the rectangular IR2 piece over the cable side of IR3, and secure both with M2 screws. Slide one IR1 spacer onto the lens (Fig. 20a).
• Optionally, drill mounting holes in IR4 to match IR3′s circuit board. Install M3 standoffs on IR3 and fasten the board to IR4 with M3 screws to relieve stress on the camera core (Fig. 20b).
• In IR5a, bore two opposing holes for the camera and drill corresponding M3 holes in IR11. Place IR4 (with camera mounted) into one hole of IR5a, secure with screws, then add a second IR1 spacer outside the lens. Thread the hexagonal IR2 piece onto the lens (Fig. 20c).
• Attach IR5a to IR11 using M3 bolts and nuts.
• Add IR6 to the adjacent hole on IR5a, connect IR7 between the USB port on IR3 and IR8. Then connect IR9 to IR8, pass it through the IR6 then tighten it and pack any gaps with foam. Place IR5b atop IR5sa and screw shut (Fig. 20d-e).
• Attach IR11 to IR12 using M6 screws.
b. Visible light camera

**Fig. 21:**
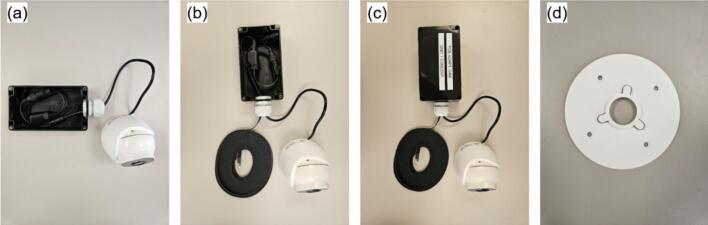
Assembly procedure of the visible light camera.

Key steps are illustrated in Fig. 21.

• Drill on single hole on one of the short edges of VL2b, install VL4, route VL1 cables through the hole, and connect VL5 to VL1 (Fig. 21a)
• Pass one end of VL3 through VL4, connect it to the opposite VL5 connector and seat VL2a atop of VL2b, and tighten VL4. Fill any voids with foam for a tight fit (Fig. 21b-c).
• Remove VL1′s bottom plate (Fig. 21d), align and attach it to VL8 using the plate’s mounting holes.
• Affix the VL8 assembly to VL7 with M6 screws, then reattach VL1 to its bottom plate to complete the setup.
c. Current sensor

**Fig. 22:**
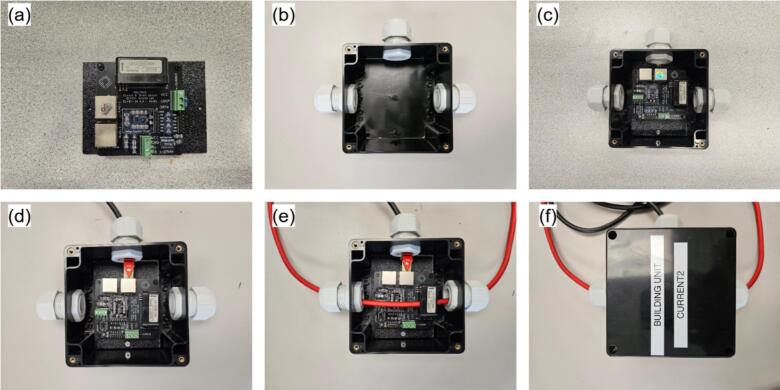
Assembly steps of the current sensor.

The current sensor assembly is depicted in Fig. 22.

• Place CS1 atop CS4 and secure with CS2 (Fig. 22a).
• Bore three openings in CS3a using a hole-saw bit and install three CS5 glands into the holes (Fig. 22b).
• Insert the CS4 into CS3a and connect cable CS6 to one RJ45 port on CS1 (Fig. 22c-d).
• Insert the electrical conductor through the adjacent CS5 gland openings and through the Hall-effect aperture in CS1 before final wiring, as the sensor is not a clamp type (Fig. 22e).
• Cover CS3a with CS3b and secure the enclosure using screws in CS2 (Fig. 22f).

### 5.4 Mounting structures

**Fig. 23:**
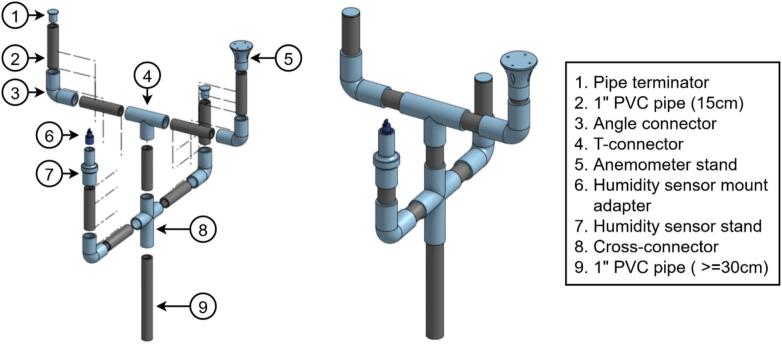
Weather station mounting structure. (Left): Exploded view. (Right): Fully assembled structure.

**Fig. 24:**
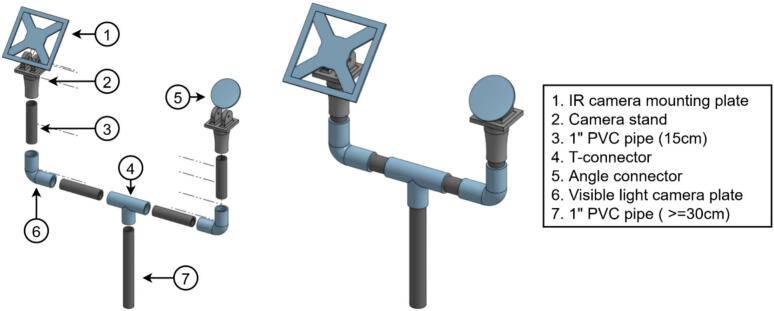
Imaging system mounting structure. (Left): Exploded view. (Right): Fully assembled structure.

Two 3D-printed-based mast assemblies support the weather-station and imaging subsystems (Fig. 23 and Fig. 24). Standard 34 mm OD pipes, cut to suit installation height, slide into matching 3D-printed connectors with a 0.5 mm tolerance for a snug fit; silicone adhesive and optional fasteners may be added for extra retention. Pipe terminators seal open ends to prevent water ingress and accommodate sensors—such as the pyranometer—which clamp around, rather than rest atop, the mast (Fig. 23a). The pyranometer needs to be installed facing south to avoid shading. After erecting the mast on a driven square or L-section base, individual sensors mount directly to their respective connector interfaces.

The camera mounting assemblies; comprising IR7, IR11, VL6, and VL7; feature adjustable plate-and-stand interfaces that permit post-installation alignment. Each pair of stand and plate incorporates mounting holes fitted with M6 butterfly wing bolts, enabling tilt adjustments without disassembly.

### 5.5 Final connections

**Fig. 25:**
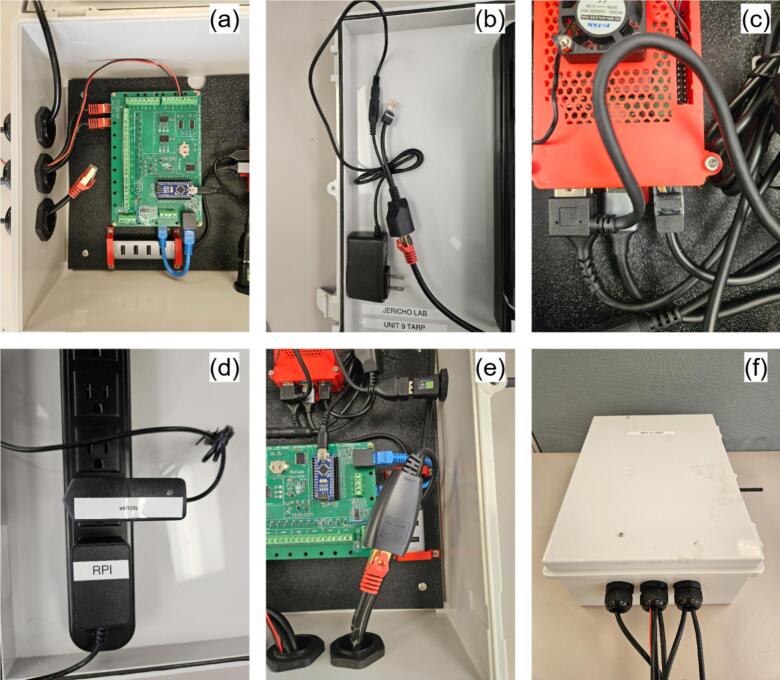
Final connections steps.
• For the visible-light camera, pass VL3 through a gland on MH1, plug into VL6, then power via the power bar and Ethernet into MH5 (Fig. 25b-c).
• For the IR camera, route IR9 through its gland to IR10 and into MH5 (Fig. 25e).
• Close the main hub and tighten all cable glands

Once all sensors are assembled and installed and in their respective use location, route and connect cables at the main hub as follows:

• Connect thermistors, followed by I^2^C devices over RJ45, then analog sensors to facilitate cable management (Fig. 25a).

**Fig. 26:**
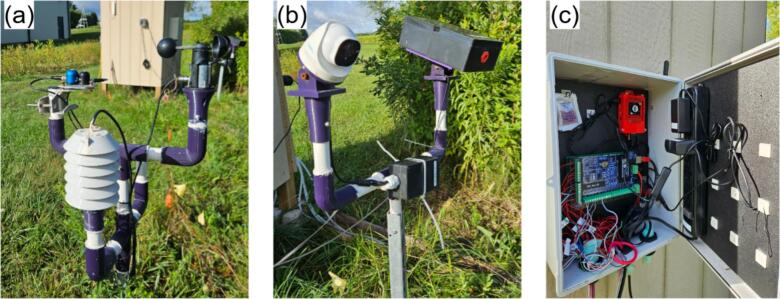
Field deployed sub-systems: (a) weather station, (b) imaging system and (c) main hub.

The field-deployed main hub, weather station, and imaging systems are shown in Fig. 26.

## 6 Operation instructions

The following procedure describes how to operate the JOR system. It assumes the user has access to a functioning laptop or desktop computer with the Arduino IDE installed, as well as a Raspberry Pi with Raspberry Pi OS, screen, keyboard, and mouse. An active internet connection is also required for full system configuration. All software and source code are provided in the supplementary files. These steps should be completed prior to final system assembly and deployment.

### 6.1 Setup Arduino code and upload to board from a laptop or desktop

**Fig. 27:**
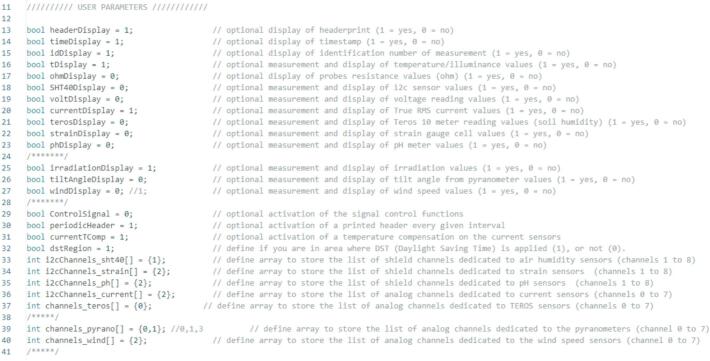
Use parameter settings in the Arduino IDE.
d. Compile the code to verify that all required libraries are available.
e. If missing, install the required libraries from the Arduino-libraries subfolder.
f. Once the code compiles without errors, upload it to the Arduino Nano and confirm proper operation using the Serial Monitor.
g. To configure the real-time clock (RTC):
  • Remove the RTC battery.
  • Disconnect the Nano from the computer.
  • Reconnect the Nano while the battery remains removed.
  • Compile and upload the code again.
  • Immediately reinsert the battery once the upload completes.
  • The RTC is now set to the most recent code compilation time.
h. The Nano is now ready for operation.

The Arduino scripts are provided in the RDL_main folder within the Source_Code package.

a. Open the EEPROM-initialization.ino file in the Arduino IDE and upload it to the Nano.
b. Open the RDL_main.ino file in the Arduino IDE.
c. Define user parameters according to the desired outputs and the number and type of sensors connected to the system (Fig. 27).

### 6.2 Install and operate Raspberry Pi

**Fig. 28:**
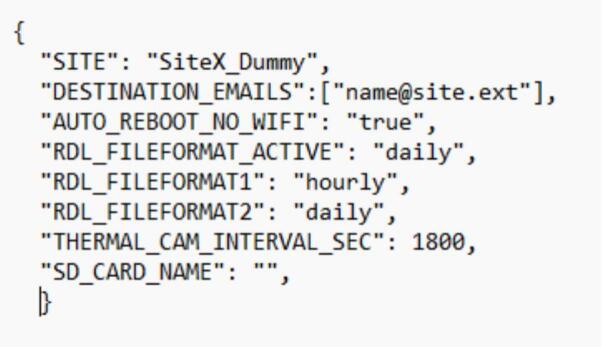
System operation configuration file.
e. Install RustDesk for remote access
  • Download the Ubuntu/AArch64 RustDesk “.deb” file from the RustDesk GitHub repository [34].
  • Run the “rustdesk-reset-install.sh” script to install RustDesk and register it as a background service. This script may also be used to reset RustDesk if issues occur. For a fresh installation, run the following in a terminal: Fig. 29.

a. Connect the Raspberry Pi to an active internet network.
b. Copy the Python-Scripts folder to a working directory on the Pi.
c. Install the required Python dependencies by executing the setup_python_libraries.sh script in the terminal.
d. Configure the system by editing the config.json file (Fig. 28).

**Fig. 29:**
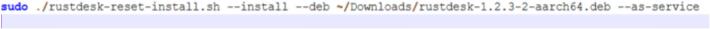
Terminal script to install or reset RustDesk.
  • Launch RustDesk and record the system ID, which can be used for remote access to the system and data transfer when the system is online.
f. Configure the visible-light camera by assigning a static IP address, username, and password using the Reolink software [39]. Update these parameters in the WIRED-logging-Reolink1.py script. (See Fig. 30.)

**Fig. 30:**
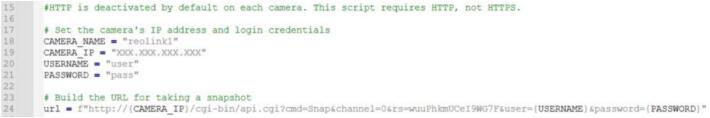
Reolink script to configure the camera’s IP address.
g. Enable the data logging daemons by navigating to the daemons-wired folder and executing wired-enable-start-all-daemons.sh. This ensures all daemons run continuously in the background, including after system reboots.
h. Verify daemon status using wired-verify-status-daemons.sh. This step should be tested with both the RDL and camera before deployment.
i. The system can now be accessed remotely using RustDesk when connected to the internet.
j. All measurement data are stored in the logging-folder directory.
k. Optionally, the Arduino IDE can be installed on the board to enable remote troubleshooting.

### 6.3 Data file structure and access

**Fig. 31:**
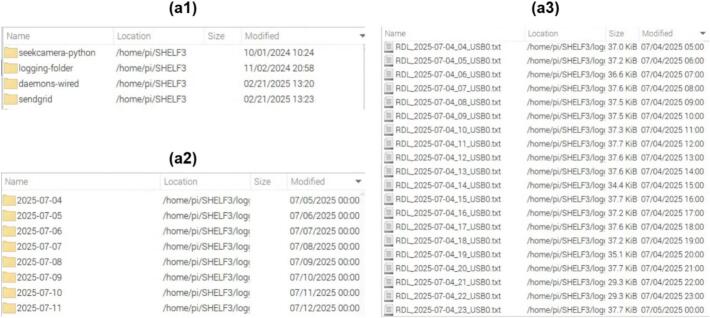
Hierarchy of files in the Python script folder showing the logged files from the RDL. (a1) Main folder. (a2) Logging folder. (a3) Logged files in a folder.

The logging folder resides under the Python-Scripts folder, named SHELF 3 in Fig. 31a, and contains daily subfolders automatically generated by the python logging script. The script first checks whether a folder for the current day exists and creates one if it does not. Each subfolder is named using the “YYYY-MM-DD” convention and stores hourly text files produced by the RDL as seen in Fig. 31b. During operation, the logging script either opens the file for the current hour or creates a new one, then appends the data received from the Nano. File names follow the format “RDL_YYYY-MM-DD_HH_USB0.txt”, where “HH” indicates the start hour of the data acquisition (Fig. 31c). A new file is initiated every hour to maintain data integrity and prevent data loss during long-term operations. Files can be retried either locally via USB or remotely through the RustDesk interface.

## 7 Validation and characterization

### 7.1 Validation methodology

The ability of the JOR to collect weather data was systematically evaluated through two complementary validation analyses. The first method compared prototype measurements against the commercial smart weather sensor Lufft WS 501 [53] reference system to assess measurement accuracy and precision, while the second evaluated inter-device performance consistency between two independent JOR units. All tests were conducted outdoors at the Environmental Sciences Western Field Station as part of Western Innovation for Renewable Energy Deployment (WIRED) outdoor experiments in Ilderton, Ontario [54]. In both validation analyses, the systems were co-located under identical environmental conditions. The JOR data were recorded at 20-second intervals, while the commercial sensor provided measurements at a 1-minute resolution. Data for the comparison with the Lufft WS 501 were collected between August 22 and August 26, 2025, whereas the inter-device comparison between two JOR units was acquired from July 4 to July 11, 2025. The validation approach relied on statistical analysis to quantify sensor performance across different temporal scales and environmental conditions.

#### 7.1.1 Error metrics and scatter plot analysis

For both validation studies, sensor outputs were aggregated and analyzed at minute and hourly scale. Standard statistical performance indicators were calculated, including root mean square error (RMSE), mean absolute error (MAE), mean bias error (MBE), and coefficient of determination (R^2^). These metrics provide complementary assessments of random error magnitude(Eq. (1)), absolute deviation (Eq. (2)), systematic bias (Eq. (3)), and explained variance (Eq. (4)), respectively [55,56]. Scatter plots were generated between the JOR and Lufft WS 501, and between two JOR units (A and B).

(1) $$\mathrm{RMSE}=\sqrt{\frac{1}{n}\sum_{i=1}^{n}{\left(P_{i}-O_{i}\right)}^{2}}$$

(2) $$\mathrm{MAE}=\frac{1}{n}\sum_{i=1}^{n}\left|P_{i}-O_{i}\right|$$

(3) $$\mathrm{MBE}=\frac{1}{n}\sum_{i=1}^{n}\left(P_{i}-O_{i}\right)$$

(4) $$R^{2}=1-\frac{\sum_{i=1}^{n}{\left(O_{i}-P_{i}\right)}^{2}}{\sum_{i=1}^{n}{\left(O_{i}-\overset{¯}{O}\right)}^{2}}$$

In Eqs. (1)-(4), $P_{i}$ represents one JOR unit measurements, $O_{i}$ represents either the Lufft or the second JOR unit measurements, and n is the sample size.

#### 7.1.2 Bland–Altman Agreement Analysis

For the comparison between JOR prototypes and the commercial Lufft WS 501 reference sensor, instruments performance was compared using Bland–Altman plots, which display the difference between paired measurements as a function of their mean value. This approach provides a better insight into measurement agreement compared to correlation analysis alone, as it reveals both constant and proportional bias while establishing meaningful limits of agreement between measurement methods [57,58]. The 95 % limits of agreement were calculated as the mean difference ±1.96 standard deviations (equation (5)), providing bounds within which 95 % of measurement differences are expected to fall.

(5) $$\sigma =\sqrt{\frac{1}{n}\sum_{i=1}^{n}{\left(\left(P_{i}-O_{i}\right)-\overset{¯}{d}\right)}^{2}}$$

In Eq. (5), $\overset{¯}{d}$ represents the mean difference and is calculated with the same formula as MBE in Eq. (3). $P_{i}$ denotes measurements from the JOR unit under evaluation, and $O_{i}$ denotes either the corresponding measurements from the Lufft or from the second JOR unit during inter-device comparison.

#### 7.1.3 Intraclass Correlation Coefficient for Inter-Device Reliability

To quantify measurement consistency between the two independent JOR prototype units, intraclass correlation coefficients (ICC) were computed using a two-way random effect, single measurement model, ICC(2,1). This statistical approach evaluates the proportion of total variance attributed to hardware differences between measurement targets versus random measurement error, providing a robust assessment of inter-device reliability [59,60]. ICC values approaching unity indicate high consistency between devices, while lower values suggest significant inter-unit variability that may require individual sensor calibration or highlight hardware design instability [61].

### 7.2 Validation results

#### 7.2.1 Comparison with a commercial weather sensing hardware

**Fig. 32:**
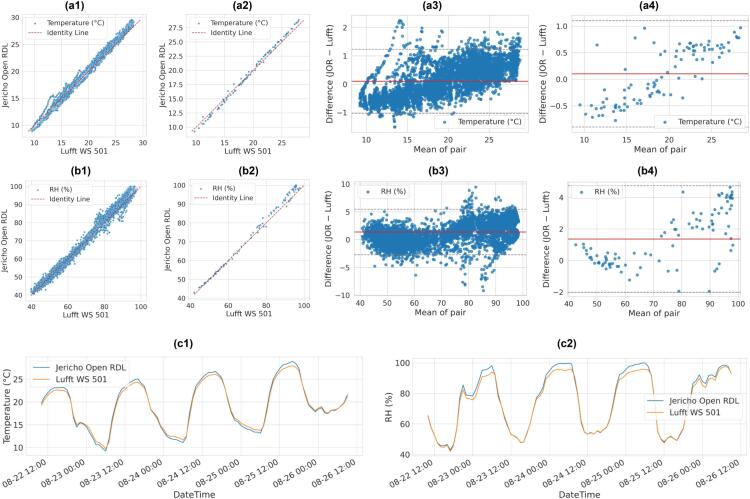
Validation of Jericho Open RDL against the Lufft WS 501 for temperature and relative humidity: (a1) Scatter plot of minutes-based temperature (°C), (a2) Scatter of hourly temperature values, (a3) Bland–Altman plot showing minute-based residuals (JOR – Lufft) vs. mean temperature, (a4) Bland–Altman plot showing hourly residuals (JOR – Lufft) vs. mean temperature, (b1) scatter plot of relative humidity (%), (b2) scatter of hourly humidity values, (b3) Bland–Altman plot showing minute-based residuals (JOR – Lufft) vs. mean RH, (b4) Bland–Altman plot showing hourly residuals (JOR – Lufft) vs. mean RH, (c1) time-series overlay of temperature over 4 days and (c2) Time-series overlay of RH over the same period.

Validation against the commercial Lufft WS 501 demonstrated that the JOR system provides research-grade measurements across the tested environmental variables. Air temperature (Fig. 32a1–a4, c1; Table 4) showed near-perfect alignment with the reference, with RMSE decreasing from 0.59 °C at the minute scale to 0.52 °C at the hourly scale. Bland–Altman analysis indicated a mean bias of +0.10 °C with 95 % limits of agreement spanning –1.0 to +1.2 °C, meaning that over 95 % of temperature differences remained within ±1 °C of the reference. Relative humidity comparisons (Fig. 32b1–b4, c2; Table 4) revealed strong correlation (R^2^ > 0.98) but a systematic positive bias (+1.37 % RH). The 95 % limits of agreement ranged from –2.7 % to +5.5 %, showing that while most measurements were within a few percentage points of the reference, JOR tended to slightly overestimate under near-saturated nighttime conditions. Hourly averaging reduced the spread and improved signal stability for both variables. (See Fig. 32.).

**Table 4:** Statistical performance of Jericho Open RDL measurements against the commercial Lufft WS 501 reference sensor.

Number of Samples	Variable	RMSE	MAE	Bias	R2	Aggregation	Subset
5760	Temperature (°C)	0.585	0.491	0.102	0.986	Minute	All
96		0.518	0.468	0.102	0.989	Hourly	
5760	RH (%)	2.495	2.004	1.366	0.982	Minute	
96		2.196	1.661	1.366	0.985	Hourly	
5760	Irradiance (W/m^2^)	76.702	25.942	-3.415	0.946	Minute	
96		12.994	7.949	-3.415	0.998	Hourly	
5760	Wind Speed (m/s)	0.600	0.513	0.318	0.446	Minute	
2208		0.553	0.432	0.068	-0.061	Minute	WS>1
96		0.450	0.327	0.318	0.617	Hourly	All
42		0.052	0.035	0.016	0.976	Hourly	WS>1

**Fig. 33:**
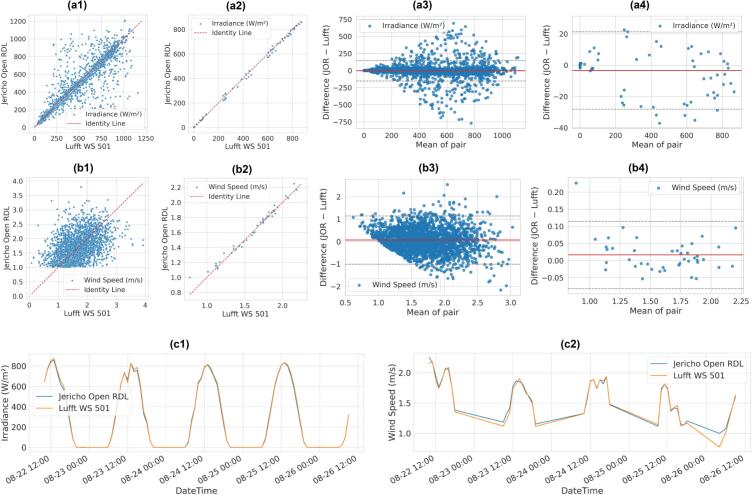
Validation of Jericho Open RDL against the Lufft WS 501 for irradiance and wind speed: (a1) scatter plot of minutes-based irradiance (W/m^2^), (a2) scatter of hourly irradiance values, (a3) Bland–Altman plot showing minute-based residuals (JOR – Lufft) vs. mean irradiance, (a4) Bland–Altman plot showing hourly residuals (JOR – Lufft) vs. mean irradiance, (b1) Scatter plot of wind speed (m/s), (b2) Scatter of hourly wind speed values, (b3) Bland–Altman plot showing minute-based residuals (JOR – Lufft) vs. mean wind speed, (b4) Bland–Altman plot showing hourly residuals (JOR – Lufft) vs. mean wind speed, (c1) time-series overlay of irradiance over 4 days, and (c2) time-series overlay of wind speed over the same period.

**Fig. 34:**
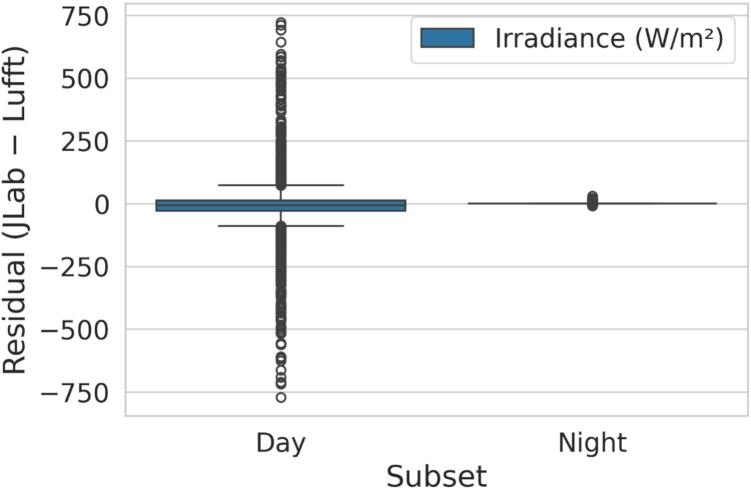
Day vs. night residuals for irradiance (JOR – Lufft)*.*

Irradiance data comparison between JOR and Lufft WS 501 (Fig. 33a1–a4, c1; Table 4) illustrates a good agreement between the sensor at a minute level, that improves with hourly aggregation. This observation is supported by R^2^ increasing from 0.95 at the minute level to 0.998 at the hourly scale. Bland–Altman plots showed a mean bias of −3.415 W/m^2^, with a measurement precision between the two systems ranging from −154 to 147 W/m^2^ at a minute-scale. These bounds reflect the natural variability of cloud transients and differences in sensor response, rather than systematic drift, as the measurement error is reduced by aggregating the data hourly (−28 to 21 W/m^2^). Residual boxplots displayed in Fig. 34 confirmed that nighttime agreement was nearly exact, while daytime scatter widened as expected under variable solar conditions.

The wind speed sensor exhibited the largest deviations (Table 4). Wind speed is displayed only for values above 1 m/s (Fig. 33b1-b4, c2) as the sensor has low accuracy at speeds below that number [62]. After filtering the data, Bland–Altman analysis showed a mean bias of 0.32 m/s, with limits of agreement of −0.3 to 1 m/s, largely driven by the stochastic nature of wind-speed fluctuations at a minute level. Aggregating the data on an hourly basis improved performance significantly, reducing the bias to 0.07 m/s and narrowing the measurement error between −0.08 to 0.11 m/s, demonstrating that the anemometer performs reliably once airflow exceeds its stall threshold (1 m/s).

#### 7.2.2 Repeatability of measurements between JOR units

**Fig. 35:**
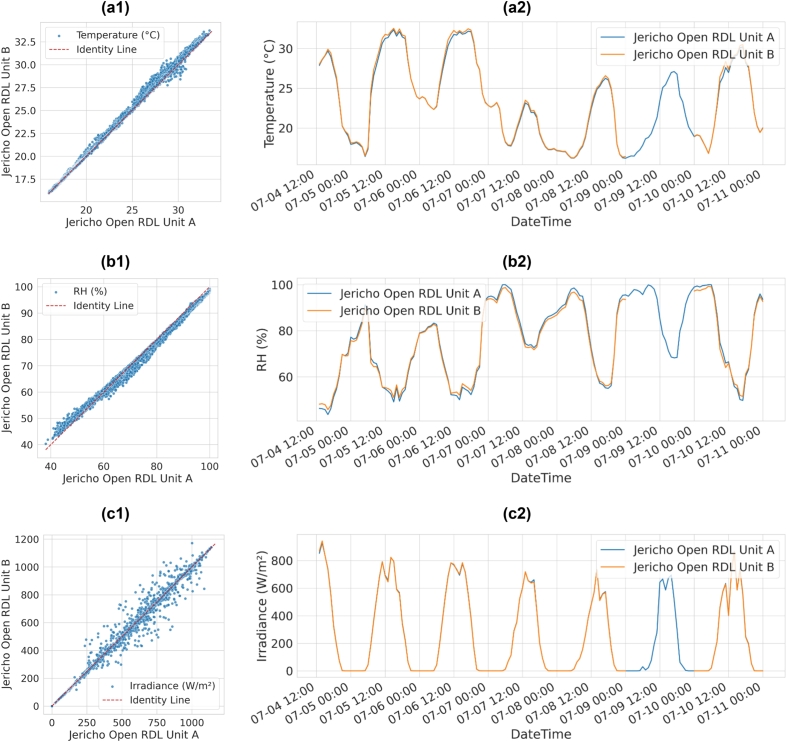
Inter-device reliability between two independent Jericho Open RDL units. (a1) Scatter of temperature (°C) between Unit A and Unit B. (a2) Time-series overlay of temperature over a week. (b1) Scatter of RH (%) between Unit A and Unit B. (b2) Time-series overlay of RH. (c1) Scatter of irradiance (W/m^2^) between Unit A and Unit B. (c2) Time-series overlay of irradiance.

Comparisons between two independent JOR units confirm excellent inter-device repeatability across all measured variables (Fig. 35a1–c2; Table 5). For temperature, scatter and time-series overlays show near-perfect alignment, with minute-level errors of RMSE = 0.30 °C, MAE = 0.20 °C, and bias = +0.19 °C, improving slightly at the hourly scale (RMSE = 0.27 °C). Relative humidity demonstrated strong agreement (minute RMSE = 1.42 %, bias = –0.39 % RH), with hourly averaging reducing errors to 1.36 %. Irradiance also showed robust consistency, with RMSE decreasing from 22.3 W/m^2^ at minute resolution to 4.0 W/m^2^ at hourly resolution, while bias remained near zero (–1.37 W/m^2^).

**Table 5:** Inter-device comparison between two Jericho Open RDL units.

Number of Samples	Variable	RMSE	MAE	Bias	R2	Aggregation
7846	Temperature (°C)	0.3041	0.2045	0.1902	0.9964	Minute
133		0.2730	0.1924	0.1887	0.9971	Hourly

7846	RH (%)	1.4246	1.2477	−0.3946	0.9931	Minute
133		1.3556	1.2282	−0.4061	0.9937	Hourly

7846	Irradiance (W/m^2^)	22.2834	6.1375	−1.3722	0.9952	Minute
133		4.0460	2.3587	−1.3139	0.9998	Hourly

Reliability metrics further confirm this consistency: R^2^ exceeded 0.993 across all variables, while ICC values remained above 0.996 regardless of time scale (diurnal or nocturnal) (Table 6). Notably, day–night stratification did not significantly alter performance; for example, temperature ICC ranged from 0.9963 (day) to 0.9997 (night), while irradiance reached 0.9999 under dark conditions. These results demonstrate that unit-to-unit variability is negligible, establishing the JOR hardware as highly reproducible for distributed deployments without requiring individual recalibration.

**Table 6:** Intraclass correlation coefficient (ICC) analysis for Jericho Open RDL inter-device reliability.

Aggregation	Subset	Variable	ICC
Minute	All	Temperature (°C)	0.9983
		RH (%)	0.9963
		Irradiance (W/m^2^)	0.9976
	Day	Temperature (°C)	0.9963
		RH (%)	0.9935
		Irradiance (W/m^2^)	0.9946
	Night	Temperature (°C)	0.9997
		RH (%)	0.9941
		Irradiance (W/m^2^)	0.9998

Hourly	All	Temperature (°C)	0.9986
		RH (%)	0.9967
		Irradiance (W/m^2^)	0.9999
	Day	Temperature (°C)	0.9969
		RH (%)	0.9943
		Irradiance (W/m^2^)	0.9997
	Night	Temperature (°C)	0.9997
		RH (%)	0.9936
		Irradiance (W/m^2^)	0.9999

### 7.3 Imaging system validation

The thermal and visible light imaging cameras integrated into the JOR system were not custom-developed hardware but standard commercial components. Unlike the RDL chain, the cameras were connected directly to the Raspberry Pi, which provides stable USB and Ethernet interfaces with well-established Linux drivers. Given the proven reliability of the Raspberry Pi for continuous video and image capture, validation efforts focused on confirming seamless integration into the data acquisition pipeline rather than characterizing the cameras themselves, which are commercially validated.

**Fig. 36:**
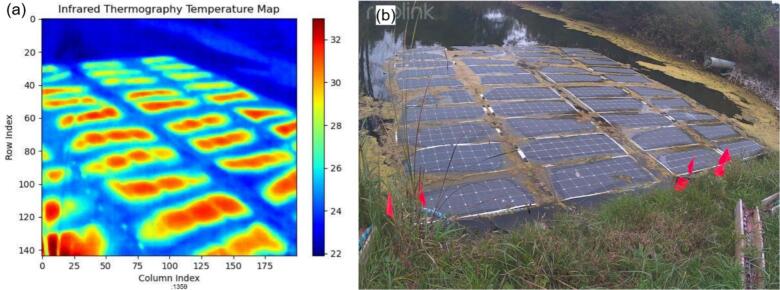
Side-by-side comparison of images captured by the thermographic camera (a) and the visible light camera (b) on October 1st, 2024, at 2:00PM.

During deployment, both cameras consistently produced stable outputs with no data loss or corruption across multi-days operation. Camera-derived data (thermal frames and contextual images) were visually inspected and found to be consistent with environmental conditions. No systematic errors were observed, confirming that the Pi-based integration approach offers reliable performance, indicating that the integration approach provides robust performance for contextual sensing within the broader JOR framework. Samples images taken by the two cameras of a foam-based floating PV system [46] are illustrated in Fig. 36.

### 7.4 Limitations and future improvements

#### 7.4.1 Hardware scalability

The current Arduino Nano controller (2 KB SRAM, 32 KB flash) restricts the number of simultaneously supported sensors, with memory exhaustion observed during multi-sensor logging. Upgrading to the Arduino Nano Every (6 KB SRAM, 48 KB flash) would triple available resources without redesign, while migration to an ESP32 platform (520 KB RAM, dual-core 240 MHz, integrated Wi-Fi/Bluetooth) could unlock substantially greater capacity. ESP32 adoption, however, would require the redesign of the RDL board due to voltage-level differences (3.3 V vs. 5 V) and alternative pinouts [63].

#### 7.4.2 Sensor accuracy

Validation revealed a consistent positive bias in relative humidity (+1–2 % RH) under high-humidity conditions and wind speed accuracy was below 1 m/s due to sensor stalling. These limitations could be addressed through sensor-specific calibration protocols or switching to different types of sensors with higher accuracy, though this would increase hardware costs. For example, humidity probes with reduced hysteresis, and low-threshold anemometers designed for calm-air sensitivity could be used [64], depending on the measurement interval and accuracy needed.

#### 7.4.3 Communication reliability

The current I^2^C backbone is limited to 30 m cable runs due to capacitive loading. Differential I^2^C extenders (e.g., PCA9600) or active bus buffers (e.g., P82B96) could extend reliable ranges to 100–300 m over Cat5e cabling, while I^2^C-to-RS485 conversion offers even greater distances and noise immunity. These approaches have been widely adopted in distributed environmental sensor networks [65].

#### 7.4.4 Remote autonomy and accessibility

For sites lacking internet access, cellular connectivity via GSM/LTE modules (e.g., SIM800L, LTE Cat-1) would enable real-time data transmission. Integration with solar charging would further allow standalone operation in off-grid contexts, as demonstrated in other low-cost wireless sensing systems achieving more than 40 days of autonomous runtime [66]. Current operation requires technical expertise to configure and interpret log files. Development of graphical user interfaces (GUIs) and web dashboards could expand usability to non-specialists. Features such as real-time visualization, automated anomaly detection, and natural-language query interfaces would improve adoption by environmental scientists, agricultural operators, and educators.

By addressing these limitations, the Jericho Open RDL system could evolve from a validated prototype into a modular platform for scalable, distributed environmental monitoring.

## Ethics statements

This work does not involve any human subjects or animal experiments.

## CRediT authorship contribution statement

**Koami Soulemane Hayibo:** Writing – review & editing. **Joshua M. Pearce:** Writing – review & editing.

## Declaration of competing interest

The authors declare that they have no known competing financial interests or personal relationships that could have appeared to influence the work reported in this paper.
